# The Therapeutic Potential of Mitochondria Transplantation Therapy in Neurodegenerative and Neurovascular Disorders

**DOI:** 10.2174/1570159X05666220908100545

**Published:** 2023-04-12

**Authors:** Mohammad Moshahid Khan, Hector G. Paez, Christopher R. Pitzer, Stephen E. Alway

**Affiliations:** 1 Department of Neurology, College of Medicine, University of Tennessee Health Science Center, Memphis, TN, 38163, USA;; 2 Neuroscience Institute, University of Tennessee Health Science Center, Memphis, TN, USA;; 3 Center for Muscle, Metabolism and Neuropathology, Division of Regenerative and Rehabilitation Sciences and Department of Physical Therapy, College of Health Professions, University of Tennessee Health Science Center, Memphis, TN, USA;; 4 Laboratory of Muscle Biology and Sarcopenia, Department of Physical Therapy, College of Health Professions, University of Tennessee Health Science Center, Memphis, TN, USA;; 5 Department of Physiology, College of Medicine, University of Tennessee Health Science Center, Memphis, TN, USA;; 6 Integrated Biomedical Sciences Graduate Program, College of Graduate Health Sciences, University of Tennessee Health Science Center, Memphis, TN, 38163, USA;; 7 The Tennessee Institute of Regenerative Medicine, 910 Madison Avenue, Memphis, TN, 38163, USA

**Keywords:** Mitochondria, mitochondrial medicine, mitochondria transplantation therapy, Alzheimer’s disease, Parkinson’s disease, stroke

## Abstract

Neurodegenerative and neurovascular disorders affect millions of people worldwide and account for a large and increasing health burden on the general population. Thus, there is a critical need to identify potential disease-modifying treatments that can prevent or slow the disease progression. Mitochondria are highly dynamic organelles and play an important role in energy metabolism and redox homeostasis, and mitochondrial dysfunction threatens cell homeostasis, perturbs energy production, and ultimately leads to cell death and diseases. Impaired mitochondrial function has been linked to the pathogenesis of several human neurological disorders. Given the significant contribution of mitochondrial dysfunction in neurological disorders, there has been considerable interest in developing therapies that can attenuate mitochondrial abnormalities and proffer neuroprotective effects. Unfortunately, therapies that target specific components of mitochondria or oxidative stress pathways have exhibited limited translatability. To this end, mitochondrial transplantation therapy (MTT) presents a new paradigm of therapeutic intervention, which involves the supplementation of healthy mitochondria to replace the damaged mitochondria for the treatment of neurological disorders. Prior studies demonstrated that the supplementation of healthy donor mitochondria to damaged neurons promotes neuronal viability, activity, and neurite growth and has been shown to provide benefits for neural and extra-neural diseases. In this review, we discuss the significance of mitochondria and summarize an overview of the recent advances and development of MTT in neurodegenerative and neurovascular disorders, particularly Parkinson’s disease, Alzheimer’s disease, and stroke. The significance of MTT is emerging as they meet a critical need to develop a disease-modifying intervention for neurodegenerative and neurovascular disorders.

## 1 INTRODUCTION

Neurodegenerative and neurovascular disorders are characterized by the gradual and progressive degeneration of brain function and constitute a major burden of disease worldwide. Unfortunately, despite decades of research, there is no effective treatment available for these diseases. Thus, patients with neurological disorders are in urgent need of disease- modifying therapies that can slow or stop the progression of the neurodegenerative process. However, a major hurdle to the development of new therapies is the gap in our knowledge of the etiology of neurodegenerative and neurovascular disorders. The importance of the mitochondrion in the maintenance and preservation of neuronal survival and function is well established, and multiple lines of evidence suggest that mitochondrial dysfunction plays a key role in the pathogenesis of many neurodegenerative and neurovascular disorders such as Alzheimer’s disease (AD), Parkinson’s disease (PD), and ischemic stroke [1-4].

Mitochondria are membrane-bound cellular organelles which play an important role in the production of adenosine triphosphate (ATP) through oxidative phosphorylation (OXPHOS). However, OXPHOS represents a significant source of toxic endogenous free radicals as byproducts of normal cellular respiration. In addition to energy generation, mitochondria play a crucial role in a myriad of physiological functions, including fatty acids biosynthesis, calcium buffering, and integration of signaling pathways that educate immune response, autophagy, and cell survival [5-7]. However, dysfunctional mitochondria limit energy availability and increase oxidative loads, calcium accumulation, and neuroinflammation. Mitochondria are dynamic organelle, and their functions are closely intertwined with their fission and fusion capacity, motility, positioning, and shape. Mitochondria contain their own DNA, which encodes 37 genes and 13 mitochondrial proteins, which are necessary for the proper function of the electron transport system. Neurons are particularly susceptible to mitochondrial dysfunction due to their dependence on mitochondria for ATP production and calcium buffering [8]. During pathological conditions, several signaling pathways are activated in mitochondria, including the opening of the mitochondria permeability transition pore (mPTP), cytochrome c leakage to induce programmed cell death, and mitophagy which ultimately may jeopardize neuronal function and survival. Moreover, glutamate excitotoxicity in the cellular milieu causes increased oxidative stress leading to mitochondrial dysfunction, which would then ultimately lead to vascular disturbance and cellular dysfunction [9, 10]. Several works have provided evidence that mitochondrial dysfunction is central in the pathogenesis of the broad spectrum of neurological disorders, such as AD, PD, and stroke [8, 11]. Therefore, therapeutic strategies targeting mitochondrial dysfunction have the potential to slow or delay the progression of neurological disorders. Given the role of mitochondrial dysfunction in neurodegenerative and neurovascular disorders, there has been considerable interest in developing therapies that can alleviate mitochondrial abnormalities and render neuroprotective effects. For instance, the neuroprotective effect of physical exercise [12-14] and pharmacological therapeutic approaches [15-17] have been examined to improve mitochondrial function in experimental models. Moreover, several clinical trials of mitochondria-targeted therapy have been conducted, but none have produced encouraging outcomes in slowing the disease progression. Therefore, a new paradigm of Mitochondrial Transplantation Therapy (MTT), based on an organelle delivery strategy which involves the supplement of healthy mitochondria to replace the damaged mitochondria, has shown promise as a treatment for neurological disorders [18-22]. The transplantation of mitochondria isolated from diverse sources, including adipose, liver, and muscle, has been shown to cross the blood-brain barrier (BBB) and help alleviate multiple CNS diseases, including PD, AD, and ischemia/reperfusion injury (Table **1**) [23-29]. Moreover, they are readily internalized into various tissues and cells, making them more versatile in delivery [23, 30]. MTT suppressed brain inflammatory response, limited oxidative stress and improved depression-like behaviors in a rodent model of depression [31]. The early clinical feasibility of MTT in humans has provided hope for alleviating genetic mitochondrial disorders [32], and MTT has been shown to enhance post-ischemic functional recovery and reduce infarct size [33-35]. Findings from recent studies suggest that mitochondria are released into extracellular spaces and are transferred between cells in the central nervous system (CNS) [20, 36, 37].

Interestingly, MTT is a rapidly growing area of focus in the neuroscience research field. Its potential as a novel therapy is evident from the exponential increase in the number of published articles on MTT as searches that were completed on PubMed, MTT and neurological disorders showed 240 articles that were published in a period from 2000 to 2015. Evidently, MTT in neurological disorders has received more attention, with 267 articles on PubMed in the period from 2015 to 2022. These studies led to the emerging concept that MTT is a potential and feasible therapeutic approach to neurodegenerative and neurovascular disorders. However, MTT must still be explored for its safety and mechanism of therapeutic benefits. In this review, we assess mitochondrial dysfunction in neurodegenerative and neurovascular disorders and discuss the recent advances in MTT as a novel and promising treatment for neurodegenerative and neurovascular disorders.

## 2 SIGNIFICANCE OF MITOCHONDRIA IN HEALTH AND NEURODEGENERATIVE AND NEUROVASCULAR DISORDERS

Mitochondria are double-membrane cellular organelles and are often known as the powerhouse of the cell because they are the primary unit for generating ATP through OXPHOS. Although they are known to create energy for a cell to function, mitochondria also regulate multiple cellular processes involved in survival and death, lipid biogenesis, calcium buffering, cellular signaling, and degradation of misfolding proteins [5-7, 38]. The human brain constitutes nearly 2% of total body weight but accounts for 20% of the total body energy consumption; therefore, functional mitochondria are indispensable to meet the energy demand of a healthy brain. In neurons, mitochondria are required for energy metabolism and calcium buffering, which are fundamental for normal cellular processes, neurotransmission, and plasticity [8, 39]. Mitochondria have also recently been implicated as a hub for the production of metabolic intermediates that influence cellular epigenetics [40, 41]. Depending on cellular function and environmental stress, mitochondria can adopt different sizes, shapes, and numbers. Mitochondria consists of two membranes, the outer membrane and the inner membrane with folded cristae. The inner membrane of mitochondria contains the complexes of the OXPHOS and electron transport system and serves as the most active sites for cellular metabolism. Mitochondria contain their own DNA (mtDNA), which encodes 13 proteins for the electron transport chain and several tRNAs. Together with mitochondrial proteins encoded by the nuclear genome, they maintain mitochondrial health [21, 42]. The maternal inheritance of mitochondria is thought to contribute to the higher incidence of disease transmission from mothers to their progeny, which has been linked to dysfunctions in mitochondrial respiration and glucose metabolism. During metabolic distress or pathological conditions, several pathways are activated in mitochondria, including the opening of the mPTP, cytochrome c release, activation of cell death pathways, and mitophagy [8]. Cytochrome c released into the cytoplasm binds to apoptosis-activating factor-1 to form the apoptosome, which initiates the caspase cascade leading to apoptosis [43]. Other than apoptosis, there are several other non-apoptotic pathways, including necroptosis, pyroptosis, parthanatos, and ferroptosis, all of which have been related to mitochondria [8, 44].

Mitochondrial dynamics play an important role in ensuring mitochondrial homeostasis and quality and are tightly regulated by the fusion and fission machinery. Mitochondria fusion is mediated by the GTPases Opa1 and Mitofusin-1 (Mfn1) and Mfn2, whereas fission is mediated by the mitochondrial fission 1 protein (Fis1), mitochondrial fission factor (Mff), and dynamin-related protein (Drp1). Impaired mitochondrial dynamics can lead to abnormal transport and distribution of mitochondria in neurons and can negatively impact synaptic and neuronal function [45]. Mitophagy plays an essential role in the removal of damaged or dysfunctional mitochondria and maintaining steady mitochondrial turnover [46, 47], which is essential for maintaining cellular homeostasis. Consistent with this notion, impairments in mitophagy cause an accumulation of dysfunctional mitochondrial, which in turn increase oxygen consumption and reactive oxygen species (ROS) production, and eventually lead to cell death [46, 48]. Given the significance of mitochondria in health and disease, we review mitochondrial dysfunction and altered mitochondrial dynamics in human neurodegenerative and neurovascular disorders such as Alzheimer’s disease, Parkinson’s disease, and stroke.

### 2.1 Mitochondrial Dysfunction in Alzheimer’s Disease

Alzheimer’s disease (AD) is the most common form of age-associated dementia. Nationwide, the incidence of AD and care costs is rising nearly exponentially. The medical cost for AD is expected to be $ 290 billion in 2019, and caregivers will provide an estimated 18.5 billion hours in unpaid care valued at $234 billion [49]. AD is characterized by memory deficits and cognitive decline associated with neuropathological findings of neurofibrillary tangles and amyloid plaques. These characteristics are further accompanied by synaptic dysfunction, neuroinflammation and progressive neurodegeneration. Despite gaining considerable knowledge about the pathological mechanisms of AD over the last several decades, there are no disease-modifying drugs available for AD only symptomatic treatments *via* modulating neurotransmitter disturbance. Decades of research on rodents and humans showed that mitochondrial dysfunction plays a significant role in AD [50-54]. The mitochondrial cascade hypothesis argues that mitochondrial dysfunction is the primary process that triggers the deleterious cascade of events that lead to sporadic late-onset AD [55]. In support of this idea, several previous studies documented the causal role of mitochondrial dysfunction in AD pathology [52, 56, 57].

Glucose utilization in the brain is broadly used as a primary measure to determine energy metabolism. PET imaging studies have demonstrated a significant decline in glucose utilization in brain regions involved in learning and memory of AD patients as compared to controls [58, 59]. The results of these studies are further supported by findings of reduced glucose metabolism in the brains of young adults harboring the apoE4 allele several decades before the appearance of the neuropathological symptoms [60]. Several characteristics of mitochondria, such as morphology and number, oxidative phosphorylation, calcium buffering, ROS production, mtDNA oxidation and mutation mitochondrial biogenesis, mitochondria dynamics and mitophagy were found to be compromised in AD [39, 61-64]. For instance, reduced OXPHOS complex activities and impaired activities of several enzymes involved in mitochondrial energy production have been reported in postmortem brain tissue from patients with AD [39, 65-67]. Oxidative stress is caused by an imbalance between the production of ROS and antioxidants in the cellular milieu. During normal physiological conditions, mitochondrial metabolism inevitably produces ROS, which can accumulate if not adequately buffered and have been suggested to merely cause cellular damage [68]. Mitochondria are susceptible to oxidative damage, and increased ROS accumulation can lead to mitochondrial DNA damage, disrupt the mitochondrial respiratory chain, impair membrane permeability, disturb calcium homeostasis, and ultimately cause neuronal dysfunction [69, 70]. These findings suggest that oxidative stress and mitochondrial dysfunction are closely intertwined and play a critical role in the aging and age-related neurological disorders such as AD [70, 71]. Interestingly, supplementation with antioxidants can improve cognitive function and prevent AD-related neuropathology in preclinical models of AD [72-74], suggesting the deleterious role of oxidative stress and mitochondrial dysfunction in AD conditions. Mitochondrial biogenesis plays an important role in preserving mitochondrial homeostasis to meet the physiological demands of cells. PGC-1α is the master regulator of mitochondrial biogenesis and regulates the expression of genes involved in energy homeostasis through interactions with different transcription factors, including nuclear respiratory factor 1/2. Reduced expression levels of proteins regulating mitochondrial biogenesis were observed in human AD brains and experimental models of AD [63, 75, 76]. Mitochondria are highly dynamic cellular organelles that continuously fuse and divide in highly regulated but opposing manners. Mitochondrial morphology and dynamics are critical to normal cellular function, and impaired balance of the mitochondrial fission-fusion process leads to mitochondrial dysfunction and neurodegeneration in AD [77-79].

Mitochondria develop a sophisticated mitochondrial quality control system to manage inevitable damage to its contents or superfluous mitochondria. At the organelle level, damaged mitochondria are degraded by a selective form of autophagy which is termed mitophagy. Growing evidence suggests that mitophagy function is impaired in AD, resulting in the accumulation of dysfunctional mitochondria and promotes Alzheimer’s related neuropathology and cognitive deficits [80, 81]. Interestingly, mitophagy enhancement reduces Alzheimer’s pathology and ameliorates memory impairment [80, 82]. As discussed throughout, mitochondrial dysfunction is a prominent aspect of AD pathology and thus represents promising therapeutic targets.

### 2.2 Mitochondrial Dysfunction in Parkinson’s Disease

Parkinson’s disease (PD), a progressive neurodegenerative disease, is characterized by the death or malfunction of dopaminergic neurons in the substantia nigra and dopamine depletion in the striatum resulting in loss of motor functions [83, 84]. Pathologically, the hallmark feature of PD is the presence of abnormal filamentous aggregates called Lewy bodies, which are composed of the α-synuclein protein. Several hypotheses have been proposed to explain the cause of PD, of which mitochondrial dysfunction plays a central role both in sporadic and familial PD [85-88]. Past studies have documented defective mitochondrial complex I activity in the brains, as well as in the platelets, lymphocytes, and skeletal muscle tissue obtained from PD patients [85, 89, 90]. Considering this fact, mitochondrial complex I inhibition with neurotoxins rotenone and MPTP produce neuropathologic and behavioral symptoms in animal models, similar to human PD cases. Based on the premise that mitochondrial dysfunction triggers the loss of dopaminergic neurons, studies from experimental models and human PD provide strong evidence for disruptions in mitochondrial dynamics such as fusion or fission [91, 92], bioenergetics defects [93, 94], mitochondrial DNA deletion [95], complex I inhibition [96], changes in size and morphology [97], alterations in trafficking or transport [85], altered movement of mitochondria [98], and increased oxidative stress [71, 85, 90]. Increased ROS production further damages the mitochondrial genome and components of the mitochondrial respiratory chain and triggers a vicious cycle between mitochondrial dysfunction and oxidative stress [88, 99]. Moreover, mutations in several genes that cause familial PD, such as PINK1, Parkin, LRRK2, and DJ-1, are directly or indirectly involved in the regulation of mitochondrial homeostasis [100-103]. Recent studies indicate that α-synuclein, a pathological hallmark of PD, can interact with mitochondria by binding to the outer mitochondrial membrane [104]. The interaction of α-synuclein oligomers with mitochondrial membranes is closely correlated with the reduction in mitochondrial function and neuronal dysfunction [105]. The significance of mitochondria in PD is further supported by the finding that showed the beneficial effects of deep brain stimulation on energy production and mitochondrial function [106, 107]. These findings provided proof-of-concept evidence that impairments in mitochondrial function can render the brain more vulnerable to PD-related pathologies and strongly endorse the hypothesis that restoring mitochondrial function will be an important therapeutic strategy for PD.

### 2.3 Mitochondrial Dysfunction in Stroke

Stroke is the most common cause of serious disability for adults and the leading cause of death worldwide. According to the World Health Organization (WHO), approximately 15 million people worldwide suffer from stroke each year. Of these, more than 5 million die and another 5 million are left permanently disabled, placing a huge burden on the family and the community [47]. To date, recombinant tissue plasminogen activator (tPA) is the only FDA approved recommended for the treatment of ischemic stroke. However, the efficacy of tPA is limited within 4.5 h after stroke onset and therefore, not all stroke patients can receive it in a timely manner. Therefore, there is an unmet need to develop effective therapeutic options for stroke patients. Most of the stroke cases are ischemic in nature, which accounts for about 87% of cases. Mitochondrial dysfunction has been recognized as one of the hallmarks of ischemic stroke and contributes to the pathology of ischemic stroke [108, 109]. Mitochondria play a critical role in maintaining cellular energy and regulating calcium homeostasis and they are essential in enhancing neuronal survival, and neurological improvement following ischemic strokes. During an ischemic condition, a sudden reduction of regional cerebral blood flow leads to the deprivation of glucose and oxygen and perturbed cellular homeostasis, which triggers diverse pathophysiological mechanisms, including excitotoxicity, oxidative stress, mitochondrial dysfunction, calcium overload, neuroinflammation, and apoptotic cell death [110-113]. It is suggested that mitochondrial dysfunction following ischemic stroke leads to apoptotic cell death through depletion of ATP, overwhelming ROS generation, calcium overload, mPTP opening, cytochrome c releasing and impairment in mitochondria quality control machineries [4, 114-119].

During reperfusion, blood flow may be restored in time to maintain neuronal viability; however, reperfusion can itself induce mitochondrial to produce substantial amounts of ROS [120]. Mitochondrial depolarization in response to the ischemic condition drives excessive ROS production and ATP depletion [117]. While ATP paucity in ischemic cells compromises the cell membrane and renders it more permeable to the passage of sodium, chloride and water from the extracellular space [121, 122], ROS overproduction causes both functional and structural damage in the brain. Therefore, maintaining mitochondrial function is essential for improving neuronal survival and function after an ischemic stroke.

The B-cell lymphoma (BCL-2) protein family, a major regulator of the outer mitochondrial membrane, plays an important role in the regulation of neuronal death in ischemic stroke [47, 123]. Additionally, evidence from recent studies suggests that mitochondrial dynamics play a critical role in the regulation of cell survival and death after ischemic stroke [4]. To this end, mitochondrial fission is detrimental to neurons, while mitochondrial fusion allows damaged mitochondria to be repaired during ischemic conditions. Dynamin-related protein 1 (Drp1), a key regulator of fission, plays an important role in ischemic stroke [124], and Drp1 suppression reduces infarct volume following ischemic stroke [125]. Exercise preconditioning decreased cerebral edema and improved neurologic function in ischemic stroke through upregulation of mitochondrial fusion protein OPA1 [126]. Thus, it is reasonable to infer that limiting uncontrolled mitochondrial fission and increasing mitochondrial fusion, and thereby maintaining the equilibrium of mitochondrial dynamics, could be an effective therapeutic approach for the treatment of ischemic stroke. Emerging evidence obtained from several recent studies suggests that mitophagy plays critical roles in maintaining mitochondria homeostasis by removing damaged mitochondria after ischemic stroke [48, 127]. Consistent with this notion, modulating mitophagy by pharmacological compounds or exercise to remove dysfunctional mitochondria or prevent apoptotic signaling pathways has been shown to be beneficial in ischemic stroke, as reported by several studies [48, 128-130]. Thus, preserving mitochondrial integrity through regulation of mitochondrial dynamics, biogenesis and clearance of damaged mitochondria are important avenues to prevent mitochondrial dysfunction and neuronal death after ischemic stroke.

## 3 MITOCHONDRIA-BASES THERAPIES FOR NEURODEGENERATIVE AND NEUROVASCULAR DISORDERS

Mitochondrial medicine refers to disease-modifying intervention for diseases or conditions caused by mitochondria dysfunctional. Mitochondrial medicine attempts to mitigate cellular dysfunction through a targeted approach to ameliorate mitochondrial dysfunction through strategies such as increasing the capacity to generate mitochondrial ATP, reducing excessive ROS overproduction, or improving the mitochondrial quality control system. Due to the significant contribution of mitochondrial dysfunction in neurodegenerative and neurovascular disorders, there is growing interest in the development of pharmacological tools to restore mitochondrial function in preclinical models of neurodegenerative and neurovascular disorders. Unfortunately, therapies that target specific components of mitochondria or oxidative stress pathways are largely unsuccessful or failed to exhibit a protective effect in clinical studies. For example, mitochondrial biogenesis plays a crucial role in the maintenance of the mitochondrial pool and activation of PPARγ increases mitochondrial biogenesis. To this end, the effect of several PPARγ agonists such as pioglitazone and rosiglitazone that increase mitochondrial biogenesis have been tested in mouse models of AD [131, 132], PD [133], and stroke [134, 135]; however, their efficacy has come into question after human studies [136, 137]. Among drugs tested, clinical trials to rescue mitochondrial function using creatine or co-enzyme Q10 in manifest PD have been terminated due to lack of efficacy [138]. Similarly, although the neuroprotective effect of several mitochondria-targeted antioxidants such as MitoQ, MitoApocynin, and MitoTEMPO have been reported in experimental models of these neurological disorders [139-142], these results are either rarely translated into clinical trials or did not produce encouraging outcome [143]. The free-radical trapping agent NXY-059 reduced brain damage and showed promise as a neuroprotectant in an animal model of stroke. However, it was found to be ineffective in double-blinded large-scale clinical trials for the treatment of stroke [144]. The efficacy of isradipine to block calcium channels and attenuate the production of mitochondria ROS [145] has come into question after a recent STEADY-PD Phase III clinical trial. Inosine, a urate precursor and potential antioxidant, was shown to prevent dopaminergic neuronal loss in the experimental models [146]. The SURE-PD3 Phase III clinical trial is ongoing with pending results. Growing evidence from several preclinical studies has shown that targeting mitochondrial quality control and mitochondrial dynamics *via* pharmacological compounds or genetic approach are potential therapeutic approaches which render neuroprotective effects. For example, Drp1 inhibition reduces mitochondrial fission and preserves normal mitochondrial morphology and function during neurological conditions [147-149]. While aberrant mitophagy is associated with neurodegeneration, mitophagy-inducing agents have been shown to be neuroprotective in an experimental model of neurological disorders [128, 150, 151], indicating proper mitochondrial clearance is necessary for neural health. However, the mechanisms by which mitophagy enhancing compounds confer therapeutic benefits in clinical trials are unknown. We speculate that the failure of the clinical trials targeting mitochondria in neurological disorders may be due to the complexity of mitochondrial dysfunction, and thus targeting a single aspect of mitochondrial dysfunction may not be sufficient. To overcome this issue, a novel therapeutic approach has recently emerged that involves transplantation of healthy donor mitochondria into host damaged tissue to restore cellular homeostasis.

### 3.1 Mitochondrial Transplantation Therapy and Challenges

Mitochondrial transplantation therapy (MTT) has received increasing attention over the past few years because it presents a novel perspective of mitochondria medicine for several neural and extraneural diseases [8, 18, 19, 22, 33, 36, 152]. The concept of mitochondrial transplantation was conceived from the observation that mitochondria could be transferred between cells through several mechanisms, including the formation of tunneling nanotubes, extracellular vesicles, gap junctions, and cell fusion/mitochondrial extrusion [153, 154]. Consistent with this notion, the co-culture of healthy cells with cells containing dysfunctional mitochondria results in the transfer of healthy mitochondria into defective cells [155]. Several previous studies have shown the efficacy of MTT in co-culture as well as in rodent models with significant functional outcomes. For example, the transfer of mitochondria from mouse bone marrow derived stromal cells (BMSCs) has been shown to protect pulmonary alveoli against lipopolysaccharide (LPS)-induced acute lung injury in mice [156]. The beneficial effect of MTT was associated with its ability to increase ATP production and regulation of mitochondria homeostasis. Interestingly, BMSCs containing damaged mitochondria failed to confer protection, further endorsing the protective role of MTT. Similarly, Hayakawa and coworkers reported that astrocytes release mitochondria-containing vesicles through CD38-mediated mechanisms that enter neurons which confer neuroprotection in an experimental model of ischemic stroke [157]. By the same token, Elliot *et al*. have shown that transplantation of healthy mitochondria into human breast cancer cells limits cancer cell proliferation and increases the sensitivity of the cell line to breast cancer medication, suggesting the potential of MTT for cancer therapeutics [158]. Moreover, the clinical feasibility of MTT in humans has provided hope for treating mitochondrial disorders [32], and transplantation of mitochondria into the myocardium both of humans and animal models results in significant improvement in cardiac function following ischemia-reperfusion injury [35, 159-162]. Robicsek and colleagues showed the beneficial effect of MTT in an experimental model of Schizophrenia [163]. Using an *in vitro* approach, they showed that donor mitochondria could enter various cell types and improve impaired mitochondrial function. Using a rodent model of Schizophrenia, they further showed that intra-prefrontal cortex transplantation of isolated mitochondria ameliorated mitochondrial dysfunction in neurons and improved Schizophrenia-related deficit. Consistent with this study, MTT reduced brain inflammatory response and oxidative loads in the LPS-induced mouse model of depression [31].

The mechanisms by which MTT functions to increase cellular viability are not yet clear. The use of MTT dates back to 1982, when Clark and Shay first used isolated mitochondria from cells with mutations in the mitochondrial ribosomal RNA gene to confer antibiotic resistance in sensitive wild type recipient cells through coincubation [164]. In line with the endosymbiosis theory of mitochondrial origin, mitochondria appear to readily incorporate themselves into recipient cells as well as be transferred out of cells. Otherwise, healthy retinal ganglion neurons were also found to shed their mitochondria *via* axonal protrusions that were engulfed and digested by neighboring astrocytes, indicating that at least some portion of these mitochondria undergoes transcellular degradation *via* transmitophagy [165]. Furthermore, a potential benefit of introducing healthy donor mitochondria into cells is creating heteroplasmy within the existing mitochondrial pool with new mitochondria with healthy mitochondrial DNA (mtDNA). MTT would allow repopulation of mtDNA in cells that have accumulated mtDNA damage.

**Fig. (1):**
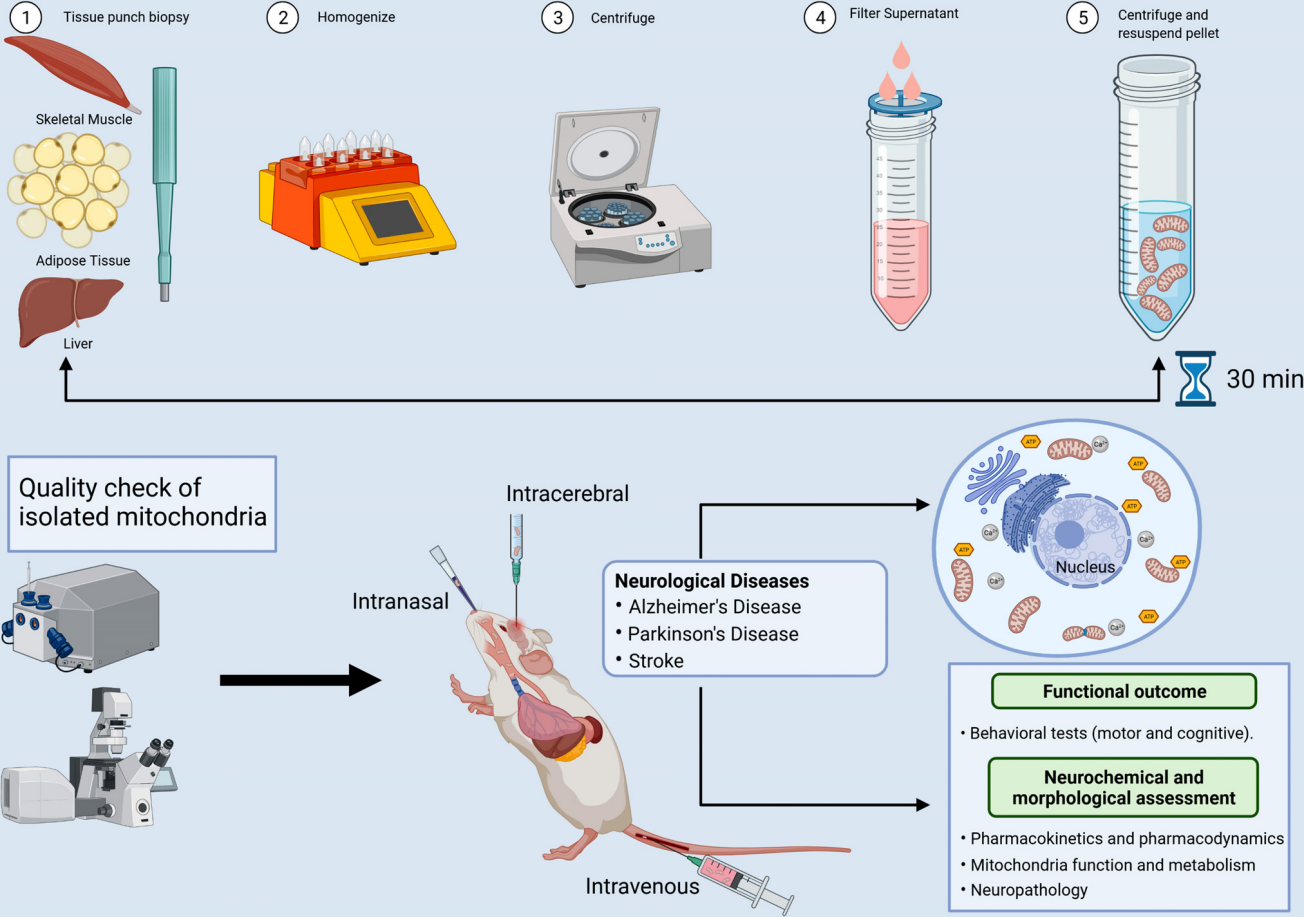
Proposed experimental approach for MTT in neurodegenerative and neurovascular disorders. Mitochondrial transplant therapy constitutes three key steps:1) isolation of healthy and functional mitochondria from diverse sources, including skeletal muscle, liver, or adipose tissue from donor species; 2) transplantation of healthy donor mitochondria into a host, either through systemic administration, intranasal delivery, or by direct injection into a region of interest in the brain; and 3) incorporation of donor mitochondria and therapeutic benefits of MTT in host target tissues/cells. Images were created with BioRender.com.

MTT is an innovative approach that consists of the following steps: 1) isolation of healthy and functional donor mitochondria from diverse sources, including adipose, liver, and skeletal muscle, or mesenchymal stem cells; 2) transplantation of the donor mitochondria to the host species, and finally; 3) incorporation of donor mitochondria and therapeutic benefits of MTT in host target tissues/cells (Fig. **1**). The ideal source of mitochondria may play an important role, as mitochondria can differ depending on their resident tissue. For example, the liver, heart, and skeletal muscle can exhibit slightly different mitochondrial proteomes [166], and it is well established that skeletal muscle contains both subsarcolemmal and intermyofibrillar populations of mitochondria with distinct biochemical and functional properties [167]. The ideal source of mitochondria for MTT may be contingent on the practical and efficient isolation of mitochondria, as MTT requires rapid isolation and administration of healthy donor mitochondria. Mitochondria derived from a liver biopsy may prove to be too invasive; however, a muscle biopsy can be performed by a trained physician and allows for transplantation of autologous mitochondria from skeletal muscle without the need for surgery. Skeletal muscle is highly abundant in mitochondria and thus may yield greater volumes of isolated mitochondria than other easily accessible peripheral tissues, such as subcutaneous adipose tissue. As a more practical means of isolating autologous mitochondria for multiple MTT, the patient’s stem cells may be collected, amplified, and stored for future mitochondria isolation and administration. Therefore, further research is necessary to elucidate if the mitochondria isolated from different sources alter their impact on recipient cells.

The therapeutic benefits of MTT depend on several variables such as, 1) the integrity and health of the donor mitochondria, 2) how many mitochondria can be taken up by cells in a certain period, and 3) how long delivered donor mitochondria remain viable and functional following transplantation. It is well known that only functional mitochondria can confer their therapeutic efficiency. Therefore, a rapid mitochondrial isolation process and quality control check of donor mitochondria are critical for optimizing outcomes. Mitochondria can be easily and rapidly isolated both from liver and skeletal muscle tissue to allow for acute use in a clinical setting in under 30 minutes, as published by the McCully research group [168]. This protocol allows for the rapid isolation of viable and morphologically intact mitochondria within a short timeframe. Moreover, mitochondria isolation kits are commercially available, indicating the feasibility of isolating mitochondria for MTT use in larger scale applications. Isolated mitochondria can be assayed for oxidative phosphorylation (OXPHOS), and only OXPHOS competent mitochondria isolates can be used for transplantation.

*In vivo*, mitochondrial transplantation can be carried out by several routes, including direct injection of donor mitochondria to target tissue, intranasal delivery, intra-arterial, or systemic delivery. Direct injections of autologous mitochondria in a porcine model of cardiac ischemia enhance myocardial cell viability and do not trigger inflammatory or immune responses [28]. In a CNS-targeted paradigm, isolated donor mitochondria can be delivered systemically *via* tail vein injections [23] or intranasal delivery [27] or direct injection to target tissue [169]. A study by Gollihue and colleagues developed an intraspinal delivery of mitochondria in a rodent model of spinal cord injury for the purpose of imaging and tracking [170]. While the above discussed MTT methods showed successful delivery of donor mitochondria into host target tissues, each method has its own limitation that requires careful consideration. The direct injection of donor mitochondria into the spinal cord or other parts of the brain and heart effectively mitigates disease progression; however, the feasibility of this approach is limited in clinical application. Given safety concerns with repeated injections, the risks may limit their use in the course of the disease condition, which would then limit therapeutic value. Therefore, it is plausible that systemic or intranasal delivery of mitochondria could be a viable strategy; however, the distribution, efficacy and safety concerns need to be further investigated.

If the MTT is carried out through a systemic route, it is also plausible that mitochondria can distribute to several organs and elicit a possible immune response. However, several recent studies suggested that the transplanted donor mitochondria do not trigger any immune or inflammatory responses [28, 171, 172]. For instance, Ramirez-Barbieri and coworkers [172] assessed the effect both of autologous and allogenic mitochondria on the immune and inflammatory responses in mice. They did not detect any immune response by single or serial injections of either syngeneic or allogeneic mitochondria in mice. Conversely, while these studies have not observed any immune response after MTT, few studies suggested that extracellular mitochondria can elicit immune and inflammatory responses [173, 174]. Considering the finding of these studies, it is critical to rigorously examine the interaction between MTT and immune response in future studies, which would be of value in terms of reducing the adverse effect associated with MTT. Another pitfall is that the quality of isolated mitochondria can rapidly degrade after isolation; thus, immediate use of isolated mitochondrial is paramount. Therefore, establishing a method that permits isolated mitochondria to be stored for an extended period would be important in expanding the MTT therapeutics. The mechanism of mitochondrial cellular uptake has been challenged, as a recent study stated that donor mitochondria were unable to withstand the ionic milieu of blood or the extracellular space [175]. However, it is possible that mitochondrial isolations in that study [175] resulted in damaged mitochondria that would be susceptible to further damage after exposure to the systemic environments. It is important to note that although studies have shown that high levels of calcium lead to mitochondrial toxicity, free viable mitochondria have been found in blood and cerebrospinal fluid [37, 176].

In order to rapidly facilitate the transfer of donor mitochondria across the plasma membrane for increasing mitochondrial intake, a new approach was established recently, where mitochondria are conjugated with cell penetrating peptides [177]. Mitochondria conjugated with cell-penetrating peptide increased mitochondrial intake and improved mitochondrial function. Similar to peptide-mediated mitochondrial delivery, Dextran, a biocompatible polymer, has been shown to protect isolated mitochondria and facilitate their cellular internalization [178]. Synaptosome-mediated mitochondria delivery restores mitochondrial function in neuronal cells containing damaged mitochondria [179]. Although these approaches could enhance mitochondrial cellular intake, these are still in the initial stage of development, and further comprehensive studies will be warranted for the evaluation of these approaches in preclinical settings.

### 3.2 Mitochondrial Transplantation Therapy is a Potential Strategy for the Treatment of Neurodegenerative and Neurovascular Disorders

Mitochondria dysfunction is an important component of neurodegenerative and neurovascular disorders such as Alzheimer’s disease, Parkinson’s disease, and stroke. Given the critical role of mitochondria dysfunction in disease onset and progression and the therapeutic setbacks of current mitochondria-based therapy for neurodegenerative and neurovascular disorders, growing attention points to the mitochondria replacement strategy. To this end, mitochondrial transplantation therapy is an alternative approach to organelle-based therapy used in mitochondrial medicine that involves the use of healthy mitochondria to replace dysfunctional or damaged mitochondria (Fig. **1**). Although the majority of research on mitochondrial transplantation has been reported in experimental models and in patients with cardiac injury to improve the cardiac function, there is growing evidence that mitochondrial transplantation is a feasible strategy to improve cellular functions in several preclinical models of human age-related neurodegenerative and neurovascular disorders (Table **1**). However, the precise mechanisms involved in observed benefits remain unclear. Supplementing healthy mitochondria to damaged neurons promote neuronal viability, activity and neurite growth and has been shown to be effective in treating neural and extra-neural diseases [29, 35, 180]. Therefore, mitochondrial transplantation therapy is an effective and feasible approach for the treatment of several neurodegenerative and neurovascular disorders in which conventional therapies are either ineffective or proved unsuccessful.

### 3.3 Mitochondrial Transplantation Therapy in Alzheimer’s Disease

Currently, there are no disease-modifying drugs available for AD. The Food and Drug Administration (FDA) has approved acetylcholinesterase inhibitors galantamine, donepezil, and rivastigmine or NMDA receptor antagonist memantine, which can simply relieve the symptoms. Although the recent FDA approved the drug Aduhelm, which has been shown to directly target the amyloid beta (Aβ) pathology of AD, its efficacy is still under debate.

Targeting the mitochondrial associated impairments may provide a favorable strategy due to the involvement of mitochondria in several AD-related pathologies, including oxidative stress, energy imbalance, and interaction with

Aβ and phosphorylated tau protein. However, several mitochondria-based therapies have been tested for murine models of AD but failed to exhibit similar neuroprotective effects in clinical studies. To overcome these limitations, MTT represents an innovative organelle-based therapy to replace dysfunctional mitochondria, thereby improving energy generation, suppressing excessive ROS production, and restoring mitochondrial function.

Supplement of healthy mitochondria into cells containing damaged mitochondria was found to be beneficial. Zhao *et al*. showed that systemic administration of the youthful mitochondria in aged mice reduced oxidative stress, enhanced mitochondrial function, and improved cognitive and motor function in aged mice [25]. Consistently, a recent study demonstrates that MTT increases mitochondrial function in the brain of aged mice through up-regulation of the mitochondrial complex II protein subunit SDHB [181]. By the same token, the intravenous administration of donor mitochondria obtained from HeLa cells ameliorated neuronal loss and reactive gliosis and improved memory deficits in AD mice [24]. In this study, donor mitochondria were detected only in the liver, despite having a beneficial effect in the brain. It is possible that the neuroprotective effect can be mediated by a cell's nonautonomous mechanism. A recent study by Bobkova and coworkers demonstrated that intranasal administration of donor mitochondria isolated from brain tissues of NMRI mice led to the improvement of spatial memory in the olfactory bulbectomized mice with Alzheimer’s type degeneration [182]. Consistent with this finding, recently, it has been demonstrated that nasal administration of mitochondria isolated from human mesenchymal stem cells effectively reversed the chemotherapy-induced cognitive deficits and restored brain health in mice [26]. Thus, mitochondria transplantation may be a promising therapeutic approach for the treatment of age-related neurodegeneration and cognitive deficits.

### 3.4 Mitochondrial Transplantation Therapy in Parkinson’s Disease

Mitochondria are potential targets to slow down or reduce PD because mitochondrial dysfunction plays a central role both in sporadic and familial PD. Given the importance of mitochondrial function in PD pathogenesis, mitochondria-based therapy that replaces dysfunctional mitochondria with healthy donor mitochondria is a potentially impactful approach to counter PD. To this end, several studies examined the efficacy and feasibility of mitochondrial delivery in reducing PD progression (Table **1**). For example, Chang *et al*. demonstrated that mitochondria conjugated with peptide infusion in the 6-hydroxydopamine (6-OHDA)-treated PC12 cells restored mitochondrial function, prevented cell toxicity, and enhanced neurite growth [169]. Intracerebral injection of donor mitochondria from allogeneic and xenogeneic sources limited dopaminergic neurodegeneration and improved neuropathological features in 6-OHDA-induced rat model of PD. In another study, Shi and coworkers demonstrated that donor mitochondria improved cell viability in 1-methyl-4-phenyl-pyridinium-treated SH-SY5Y cells [23]. Even more interesting were the facts that systemic administration of mitochondria led to widespread distribution to the brain, prevented the MPTP-induced neurotoxicity and improved behavioral performance in a mouse model of PD. A recent study by Chang and coworkers demonstrated the therapeutic feasibility of intranasal delivery of allogeneic mitochondria in a neurotoxin-induced PD rat model [27], suggesting mitochondrial delivery from the nose to the brain is a feasible approach and limits the risk associated with direct brain injection. These studies suggest that MTT may slow PD progression and demonstrate that the intravenous/intranasal administration of donor mitochondria leads to widespread distribution to the brain, thus suggesting the therapeutic feasibility of MTT in PD.

### 3.5 Mitochondrial Transplantation Therapy in Stroke

Currently, the therapeutic intervention for stroke is limited owing to a restricted therapeutic time window and unsuccessful clinical translation of several neuroprotective agents. Mitochondria are an energetic hub for regulating a wide variety of cellular functions. The discovery of mitochondrial transfer from healthy astrocytes to the ischemic neurons for the purpose of ischemic injury repair and neuroprotection provides an emerging concept for designing an organelle-based therapeutic intervention in stroke [157]. To this end, MTT offers a novel paradigm of therapeutic interventions that offer therapeutic benefits for neuronal survival and function in stroke and other acute CNS injury (Table **1**). Nakamura *et al*. showed that systemic administration of placenta-derived mitochondrial reduces infarct size after cerebral ischemia-reperfusion injury in mice [183]. Interestingly, fluorescent imaging provided evidence that transplanted mitochondria get into the brain, liver, lung, kidney, and heart at 2 hours post MTT. Intracerebroventricular transplantation of the exogenous mitochondria isolated from human MSCs reduced the MCAO-induced brain damage and functional outcome in rats *via* inhibition of apoptosis and reactive glia activation [184]. Consistent with this finding, CNS mitochondria transplantation decreased brain infarct volume, reduced cellular oxidative stress, and reversed neurological deficits after ischemia-reperfusion injury [29]. Similarly, intracerebral and intra-femoral transplantation of donor mitochondria improved the functional outcome and decreased the infarct size [185]. One important finding of this study is that disrupting electron transport or ATPase synthase in mitochondria with antimycin A and oligomycin significantly limited the neuroprotective effect, suggesting that transplantation benefits require intact mitochondrial function. Another study by Xie and colleagues examined the therapeutic benefits of MTT in cellular and animal models of stroke and found that MTT improved behavioral functions and decreased infarct size [186]. Moreover, a clinical trial (NCT04998357) examining the safety of autologous mitochondrial transplant in brain ischemia is undergoing.

## CONCLUSION AND FUTURE PERSPECTIVES

From several decades, mitochondria have been considered to be an important therapeutic target for a variety of neurodegenerative and neurovascular disorders. Significant progress has recently been made in the development of MTT as a therapeutic strategy to improve mitochondrial function in the brain. To this end, MTT represents a novel treatment paradigm of mitochondrial transfer and replacement strategies that targets mitochondrial dysfunction in disease states. While mitochondrial transplantation between one cell to another using *in vitro* approach is investigated by several labs, there has been considerable interest in testing the therapeutic benefits of MTT in experimental models of cardiac ischemia. Considering the positive outcome of MTT in cardiac ischemia, in the last few years, there has been growing interest in determining the therapeutic benefits of MTT in several experimental models of neurodegenerative and neurovascular disorders.

Replacing damaged mitochondria with mitochondria taken from healthy tissues is shaping as a promising but as yet experimental therapy to treat neurological conditions, and new breakthroughs bring MTT closer to clinical use for treating neurodegenerative and neurovascular disorders. Current data suggest that MTT significantly improves neurological complications, and at least to this point, it does not appear to produce any adverse effects in mouse models. Although early studies in humans suggest that MTT is effective in acute injuries such as cardiac ischemia reperfusion injury, yet it is also possible that MTT may be a feasible and effective treatment for other conditions, including age-related neurodegenerative disorders. Given that mitochondria are heavily implicated in the aging process, and their dysfunction results in sustained oxidative stress, metabolic dysfunction and neurodegeneration, repeated bouts of MTT may allow for the introduction of stable and healthy mitochondrial pools to substitute the dysfunctional mitochondria. In fact, MTT significantly promotes mitochondrial function, prevents neurodegeneration, and improves behavioral functions in aging and age-related neurodegenerative diseases, endorsing the application of MTT in age-related neurodegenerative diseases. However, the exact mechanism or the full therapeutic benefits of exogenous mitochondria to treat neurodegenerative and neurovascular disorders is not known, and thus, further studies are warranted. MTT is poised as a powerful tool to recapitulate a healthy metabolic environment through improvements in mitochondrial mass, ATP production, redox state, and modulation of mitochondrial biogenesis and dynamics. The finding from recent preclinical studies encourages more comprehensive and rigorous studies to examine the distribution, safety, and precise mechanisms of therapeutic efficacy of MTT which is essential for clinical translation and the expansion of therapeutic applications for several neurodegenerative and neurovascular disorders.

## Figures and Tables

**Table 1:** MTT in experimental models of neurodegenerative and neurovascular disorders.

**Neurological Diseases**	**Experimental Model**	**Species**	**Source of Mitochondria**	**Dose of Donor ** **Mitochondria**	**Route of ** **Administration**
Alzheimer’s disease	Amyloid-β neurotoxicity Aged mice Olfactory bulbectomized mice with Alzheimer's type degeneration Cisplatin-induced cognitive deficits	WT (C57BL/6) BABL/c mice NMRI mice WT (C57BL/6)	HeLa cells Mouse liver Mouse brain Human MSCs	200 µg/mouse 5 mg/kg body weight 2 to 20 µg/mouse 170 µg/mouse	Intravenous injection [24] Intravenous injection [25] Intranasal delivery [182] Intranasal delivery [26]
Parkinson’s disease	6-OHDA MPTP 6-OHDA	Sprague-Dawley rats C57BL/6J Sprague–Dawley rats	PC12 cells and Human osteosarcoma HepG2 cells Rat livers	1.05 µg/rat 0.5 mg/kg body weight 200 µg/rat	Intracerebral injection [169] Intravenous injection [23] Intranasal delivery [27]
Stroke	Focal Ischemia Model MCAO MCAO MCAO MCAO	WT (C57BL/6) Wistar rats Sprague Dawley Rats Sprague Dawley Rats Sprague Dawley rats	Cryopreserved mouse placenta Human umbilical cord derived MSCs BHK-21 cells Pectoralis major Muscle N2a and mNSC cells	100 µg/mouse 10 µl/rat 75 µg/rat 5 × 10^6^/10 µL 180-200 μg/rat	Intravenous injection [183] Intracerebral injection [184] Intracerebral injection [185] Intracerebral injection [29] Intraarterial injection [186]

## LIST OF ABBREVIATIONS

BBB: Blood-brain Barrier
BCL-2: B-cell Lymphoma
CNS: Central Nervous System
Drp1: Dynamin-related Protein 1
mPTP: Mitochondria Permeability Transition Pore
MTT: Mitochondrial Transplantation Therapy
OXPHOS: Oxidative Phosphorylation

## References

[1] Cooper J.M., Schapira A.H.V. (1997). Mitochondrial dysfunction in neurodegeneration.. J. Bioenerg. Biomembr..

[2] Moreira P.I., Zhu X., Wang X., Lee H., Nunomura A., Petersen R.B., Perry G., Smith M.A. (2010). Mitochondria: A therapeutic target in neurodegeneration.. Biochim. Biophys. Acta Mol. Basis Dis..

[3] Luo Y., Hoffer A., Hoffer B., Qi X. (2015). Mitochondria: A therapeutic target for Parkinson’s Disease?. Int. J. Mol. Sci..

[4] He Z., Ning N., Zhou Q., Khoshnam S.E., Farzaneh M. (2020). Mitochondria as a therapeutic target for ischemic stroke.. Free Radic. Biol. Med..

[5] Tait S.W.G., Green D.R. (2012). Mitochondria and cell signalling.. J. Cell Sci..

[6] Osellame L.D., Blacker T.S., Duchen M.R. (2012). Cellular and molecular mechanisms of mitochondrial function.. Best Pract. Res. Clin. Endocrinol. Metab..

[7] Angelova P.R., Abramov A.Y. (2018). Role of mitochondrial ROS in the brain: From physiology to neurodegeneration.. FEBS Lett..

[8] Norat P., Soldozy S., Sokolowski J.D., Gorick C.M., Kumar J.S., Chae Y., Yağmurlu K., Prada F., Walker M., Levitt M.R., Price R.J., Tvrdik P., Kalani M.Y.S. (2020). Mitochondrial dysfunction in neurological disorders: Exploring mitochondrial transplantation.. NPJ Regen. Med..

[9] Freire M.A. (2012). Pathophysiology of neurodegeneration following traumatic brain injury.. West Indian Med. J..

[10] Lau A., Tymianski M. (2010). Glutamate receptors, neurotoxicity and neurodegeneration.. Pflugers Arch..

[11] Johnson J., Mercado-Ayon E., Mercado-Ayon Y., Dong Y.N., Halawani S., Ngaba L., Lynch D.R. (2021). Mitochondrial dysfunction in the development and progression of neurodegenerative diseases.. Arch. Biochem. Biophys..

[12] Ma Q. (2008). Beneficial effects of moderate voluntary physical exercise and its biological mechanisms on brain health.. Neurosci. Bull..

[13] Ma C.L., Ma X.T., Wang J.J., Liu H., Chen Y.F., Yang Y. (2017). Physical exercise induces hippocampal neurogenesis and prevents cognitive decline.. Behav. Brain Res..

[14] Oliveira R.F., Paiva K.M., da Rocha G.S., de Moura Freire M.A., de Araújo D.P., de Oliveira L.C., Guzen F.P., de Gois Morais P.L.A., de Paiva Cavalcanti J.R.L. (2021). Neurobiological effects of forced swim exercise on the rodent hippocampus: A systematic review.. Acta Neurobiol. Exp. (Warsz.).

[15] Zuccato C., Cattaneo E. (2009). Brain-derived neurotrophic factor in neurodegenerative diseases.. Nat. Rev. Neurol..

[16] Weissmiller A.M., Wu C. (2012). Current advances in using neurotrophic factors to treat neurodegenerative disorders.. Transl. Neurodegener..

[17] Cao J., Hou J., Ping J., Cai D. (2018). Advances in developing novel therapeutic strategies for Alzheimer’s disease.. Mol. Neurodegener..

[18] Rabchevsky A.G., Gollihue J.L., Patel S.P. (2018). Mitochondrial transplantation strategies as potential therapeutics for central nervous system trauma.. Neural Regen. Res..

[19] Mukherjee A., Becerra C.A.D., Chavez M., Delgado J.P., Soto C. (2021). Mitochondrial transplant to replenish damaged mitochondria: A novel therapeutic strategy for neurodegenerative diseases?. Prog. Mol. Biol. Transl. Sci..

[20] Nakamura Y., Park J.H., Hayakawa K. (2020). Therapeutic use of extracellular mitochondria in CNS injury and disease.. Exp. Neurol..

[21] Chang C.Y., Liang M.Z., Chen L. (2019). Current progress of mitochondrial transplantation that promotes neuronal regeneration.. Transl. Neurodegener..

[22] Espino De la Fuente-Muñoz C., Arias C. (2021). The therapeutic potential of mitochondrial transplantation for the treatment of neurodegenerative disorders.. Rev. Neurosci..

[23] Shi X., Zhao M., Fu C., Fu A. (2017). Intravenous administration of mitochondria for treating experimental Parkinson’s disease.. Mitochondrion.

[24] Nitzan K., Benhamron S., Valitsky M., Kesner E.E., Lichtenstein M., Ben-Zvi A., Ella E., Segalstein Y., Saada A., Lorberboum-Galski H., Rosenmann H. (2019). Mitochondrial transfer ameliorates cognitive deficits, neuronal loss, and gliosis in Alzheimer’s disease mice.. J. Alzheimers Dis..

[25] Zhao Z., Yu Z., Hou Y., Zhang L., Fu A. (2020). Improvement of cognitive and motor performance with mitotherapy in aged mice.. Int. J. Biol. Sci..

[26] Alexander J.F., Seua A.V., Arroyo L.D., Ray P.R., Wangzhou A., Heiß-Lückemann L., Schedlowski M., Price T.J., Kavelaars A., Heijnen C.J. (2021). Nasal administration of mitochondria reverses chemotherapy-induced cognitive deficits.. Theranostics.

[27] Chang J.C., Chao Y.C., Chang H.S., Wu Y.L., Chang H.J., Lin Y.S., Cheng W.L., Lin T.T., Liu C.S. (2021). Intranasal delivery of mitochondria for treatment of Parkinson’s Disease model rats lesioned with 6-hydroxydopamine.. Sci. Rep..

[28] Kaza A.K., Wamala I., Friehs I., Kuebler J.D., Rathod R.H., Berra I., Ericsson M., Yao R., Thedsanamoorthy J.K., Zurakowski D., Levitsky S., del Nido P.J., Cowan D.B., McCully J.D. (2017). Myocardial rescue with autologous mitochondrial transplantation in a porcine model of ischemia/reperfusion.. J. Thorac. Cardiovasc. Surg..

[29] Zhang Z., Ma Z., Yan C., Pu K., Wu M., Bai J., Li Y., Wang Q. (2019). Muscle-derived autologous mitochondrial transplantation: A novel strategy for treating cerebral ischemic injury.. Behav. Brain Res..

[30] Kitani T., Kami D., Matoba S., Gojo S. (2014). Internalization of isolated functional mitochondria: involvement of macropinocytosis.. J. Cell. Mol. Med..

[31] Wang Y., Ni J., Gao C., Xie L., Zhai L., Cui G., Yin X. (2019). Mitochondrial transplantation attenuates lipopolysaccharide- induced depression-like behaviors.. Prog. Neuropsychopharmacol. Biol. Psychiatry.

[32] Zhang J., Liu H., Luo S., Lu Z., Chávez-Badiola A., Liu Z., Yang M., Merhi Z., Silber S.J., Munné S., Konstantinidis M., Wells D., Tang J.J., Huang T. (2017). Live birth derived from oocyte spindle transfer to prevent mitochondrial disease.. Reprod. Biomed. Online.

[33] McCully J.D., Cowan D.B., Emani S.M., del Nido P.J. (2017). Mitochondrial transplantation: From animal models to clinical use in humans.. Mitochondrion.

[34] Cowan D.B., Yao R., Akurathi V., Snay E.R., Thedsanamoorthy J.K., Zurakowski D., Ericsson M., Friehs I., Wu Y., Levitsky S., del Nido P.J., Packard A.B., McCully J.D. (2016). Intracoronary Delivery of Mitochondria to the Ischemic Heart for Cardioprotection.. PLoS One.

[35] Emani S.M., Piekarski B.L., Harrild D., del Nido P.J., McCully J.D. (2017). Autologous mitochondrial transplantation for dysfunction after ischemia-reperfusion injury.. J. Thorac. Cardiovasc. Surg..

[36] Valenti D., Vacca R.A., Moro L., Atlante A. (2021). Mitochondria can cross cell boundaries: An overview of the biological relevance, pathophysiological implications and therapeutic perspectives of intercellular mitochondrial transfer.. Int. J. Mol. Sci..

[37] Chou S.H.Y., Lan J., Esposito E., Ning M., Balaj L., Ji X., Lo E.H., Hayakawa K. (2017). Extracellular mitochondria in cerebrospinal fluid and neurological recovery after subarachnoid hemorrhage.. Stroke.

[38] Ruan L., Zhou C., Jin E., Kucharavy A., Zhang Y., Wen Z., Florens L., Li R. (2017). Cytosolic proteostasis through importing of misfolded proteins into mitochondria.. Nature.

[39] Cenini G., Voos W. (2019). Mitochondria as potential targets in Alzheimer disease therapy: an update.. Front. Pharmacol..

[40] Aon M.A., Cortassa S., Juhaszova M., Sollott S.J. (2016). Mitochondrial health, the epigenome and healthspan.. Clin. Sci. (Lond.).

[41] Xu S., Zhang X., Liu C., Liu Q., Chai H., Luo Y., Li S. (2021). Role of mitochondria in neurodegenerative diseases: from an epigenetic perspective.. Front. Cell Dev. Biol..

[42] Friedman J.R., Nunnari J. (2014). Mitochondrial form and function.. Nature.

[43] Slee E.A., Adrain C., Martin S.J. (2001). Executioner caspase-3, -6, and -7 perform distinct, non-redundant roles during the demolition phase of apoptosis.. J. Biol. Chem..

[44] Tang D., Kang R., Berghe T.V., Vandenabeele P., Kroemer G. (2019). The molecular machinery of regulated cell death.. Cell Res..

[45] Sheng Z.H. (2014). Mitochondrial trafficking and anchoring in neurons: New insight and implications.. J. Cell Biol..

[46] Guan R., Zou W., Dai X., Yu X., Liu H., Chen Q., Teng W. (2018). Mitophagy, a potential therapeutic target for stroke.. J. Biomed. Sci..

[47] Yang J.L., Mukda S., Chen S.D. (2018). Diverse roles of mitochondria in ischemic stroke.. Redox Biol..

[48] Shen L., Gan Q., Yang Y., Reis C., Zhang Z., Xu S., Zhang T., Sun C. (2021). Mitophagy in cerebral ischemia and ischemia/reperfusion injury.. Front. Aging Neurosci..

[49] El-Hayek Y.H., Wiley R.E., Khoury C.P., Daya R.P., Ballard C., Evans A.R., Karran M., Molinuevo J.L., Norton M., Atri A. (2019). Tip of the iceberg: assessing the global socioeconomic costs of Alzheimer’s disease and related dementias and strategic implications for stakeholders.. J. Alzheimers Dis..

[50] Abolhassani N, Leon J, Sheng Z, Oka S, Hamasaki H, Iwaki T (2017). Molecular pathophysiology of impaired glucose metabolism, mitochondrial dysfunction, and oxidative DNA damage in Alzheimer's disease brain.. Mech Ageing Dev..

[51] Nakabeppu Y. (2019). Molecular pathophysiology of insulin depletion, mitochondrial dysfunction, and oxidative stress in Alzheimer’s disease brain.. Adv. Exp. Med. Biol..

[52] Moreira P.I., Carvalho C., Zhu X., Smith M.A., Perry G. (2010). Mitochondrial dysfunction is a trigger of Alzheimer’s disease pathophysiology.. Biochim. Biophys. Acta Mol. Basis Dis..

[53] Manczak M., Kandimalla R., Yin X., Reddy P.H. (2018). Hippocampal mutant APP and amyloid beta-induced cognitive decline, dendritic spine loss, defective autophagy, mitophagy and mitochondrial abnormalities in a mouse model of Alzheimer’s disease.. Hum. Mol. Genet..

[54] Wang W., Zhao F., Ma X., Perry G., Zhu X. (2020). Mitochondria dysfunction in the pathogenesis of Alzheimer’s disease: recent advances.. Mol. Neurodegener..

[55] Swerdlow R.H., Khan S.M.A. (2004). “mitochondrial cascade hypothesis” for sporadic Alzheimer’s disease.. Med. Hypotheses.

[56] Nunomura A., Perry G., Aliev G., Hirai K., Takeda A., Balraj E.K., Jones P.K., Ghanbari H., Wataya T., Shimohama S., Chiba S., Atwood C.S., Petersen R.B., Smith M.A. (2001). Oxidative damage is the earliest event in Alzheimer disease.. J. Neuropathol. Exp. Neurol..

[57] Perez Ortiz J.M., Swerdlow R.H. (2019). Mitochondrial dysfunction in Alzheimer’s disease: Role in pathogenesis and novel therapeutic opportunities.. Br. J. Pharmacol..

[58] Kapogiannis D., Mattson M.P. (2011). Disrupted energy metabolism and neuronal circuit dysfunction in cognitive impairment and Alzheimer’s disease.. Lancet Neurol..

[59] Croteau E., Castellano C.A., Fortier M., Bocti C., Fulop T., Paquet N., Cunnane S.C. (2018). A cross-sectional comparison of brain glucose and ketone metabolism in cognitively healthy older adults, mild cognitive impairment and early Alzheimer’s disease.. Exp. Gerontol..

[60] Reiman E.M., Chen K., Alexander G.E., Caselli R.J., Bandy D., Osborne D., Saunders A.M., Hardy J. (2004). Functional brain abnormalities in young adults at genetic risk for late-onset Alzheimer’s dementia.. Proc. Natl. Acad. Sci. USA.

[61] Butterfield D.A., Halliwell B. (2019). Oxidative stress, dysfunctional glucose metabolism and Alzheimer disease.. Nat. Rev. Neurosci..

[62] Reddy P.H., Yin X., Manczak M., Kumar S., Pradeepkiran J.A., Vijayan M., Reddy A.P. (2018). Mutant APP and amyloid beta-induced defective autophagy, mitophagy, mitochondrial structural and functional changes and synaptic damage in hippocampal neurons from Alzheimer’s disease.. Hum. Mol. Genet..

[63] Calkins M.J., Manczak M., Mao P., Shirendeb U., Reddy P.H. (2011). Impaired mitochondrial biogenesis, defective axonal transport of mitochondria, abnormal mitochondrial dynamics and synaptic degeneration in a mouse model of Alzheimer’s disease.. Hum. Mol. Genet..

[64] Misrani A., Tabassum S., Yang L. (2021). Mitochondrial dysfunction and oxidative stress in Alzheimer’s disease.. Front. Aging Neurosci..

[65] Johri A., Beal M.F. (2012). Mitochondrial dysfunction in neurodegenerative diseases.. J. Pharmacol. Exp. Ther..

[66] Minjarez B., Calderón-González K.G., Rustarazo M.L.V., Herrera-Aguirre M.E., Labra-Barrios M.L., Rincon-Limas D.E., del Pino M.M.S., Mena R., Luna-Arias J.P. (2016). Identification of proteins that are differentially expressed in brains with Alzheimer’s disease using iTRAQ labeling and tandem mass spectrometry.. J. Proteomics.

[67] Maurer I., Zierz S., Möller H.J. (2000). A selective defect of cytochrome c oxidase is present in brain of Alzheimer disease patients.. Neurobiol. Aging.

[68] Balaban R.S., Nemoto S., Finkel T. (2005). Mitochondria, oxidants, and aging.. Cell.

[69] Butterfield D.A. (2018). Perspectives on oxidative stress in Alzheimer’s disease and predictions of future research emphases.. J. Alzheimers Dis..

[70] Patten D.A., Germain M., Kelly M.A., Slack R.S. (2010). Reactive oxygen species: stuck in the middle of neurodegeneration.. J. Alzheimers Dis..

[71] Jiang T., Sun Q., Chen S. (2016). Oxidative stress: A major pathogenesis and potential therapeutic target of antioxidative agents in Parkinson’s disease and Alzheimer’s disease.. Prog. Neurobiol..

[72] Santos J.R., Gois A.M., Mendonça D.M., Freire M.A. (2014). Nutritional status, oxidative stress and dementia: the role of selenium in Alzheimer’s disease.. Front. Aging Neurosci..

[73] Reddy P.H., Manczak M., Yin X., Grady M.C., Mitchell A., Kandimalla R., Kuruva C.S. (2016). Protective effects of a natural product, curcumin, against amyloid β induced mitochondrial and synaptic toxicities in Alzheimer’s disease.. J. Investig. Med..

[74] da Costa I.M., de Moura Freire M.A., de Paiva Cavalcanti J.R.L., de Araújo D.P., Norrara B., Moreira Rosa I.M.M., de Azevedo E.P., do Rego A.C.M., Filho I.A., Guzen F.P. (2019). Supplementation with curcuma longa reverses neurotoxic and behavioral damage in models of Alzheimer’s disease: a systematic review.. Curr. Neuropharmacol..

[75] Qin W., Haroutunian V., Katsel P., Cardozo C.P., Ho L., Buxbaum J.D., Pasinetti G.M. (2009). PGC-1alpha expression decreases in the Alzheimer disease brain as a function of dementia.. Arch. Neurol..

[76] Sheng B., Wang X., Su B., Lee H., Casadesus G., Perry G., Zhu X. (2012). Impaired mitochondrial biogenesis contributes to mitochondrial dysfunction in Alzheimer’s disease.. J. Neurochem..

[77] Hirai K., Aliev G., Nunomura A., Fujioka H., Russell R.L., Atwood C.S., Johnson A.B., Kress Y., Vinters H.V., Tabaton M., Shimohama S., Cash A.D., Siedlak S.L., Harris P.L.R., Jones P.K., Petersen R.B., Perry G., Smith M.A. (2001). Mitochondrial abnormalities in Alzheimer’s disease.. J. Neurosci..

[78] Wang X., Su B., Lee H., Li X., Perry G., Smith M.A., Zhu X. (2009). Impaired balance of mitochondrial fission and fusion in Alzheimer’s disease.. J. Neurosci..

[79] Hroudová J., Singh N., Fišar Z. (2014). Mitochondrial dysfunctions in neurodegenerative diseases: relevance to Alzheimer’s disease.. BioMed Res. Int..

[80] Fang E.F., Hou Y., Palikaras K., Adriaanse B.A., Kerr J.S., Yang B., Lautrup S., Hasan-Olive M.M., Caponio D., Dan X., Rocktäschel P., Croteau D.L., Akbari M., Greig N.H., Fladby T., Nilsen H., Cader M.Z., Mattson M.P., Tavernarakis N., Bohr V.A. (2019). Mitophagy inhibits amyloid-β and tau pathology and reverses cognitive deficits in models of Alzheimer’s disease.. Nat. Neurosci..

[81] Cai Q., Jeong Y.Y. (2020). Mitophagy in Alzheimer’s disease and other age-related neurodegenerative diseases.. Cells.

[82] Cen X., Chen Y., Xu X., Wu R., He F., Zhao Q., Sun Q., Yi C., Wu J., Najafov A., Xia H. (2020). Pharmacological targeting of MCL-1 promotes mitophagy and improves disease pathologies in an Alzheimer’s disease mouse model.. Nat. Commun..

[83] DeMaagd G., Philip A. (2015). Parkinson’s disease and its management: part 1: disease entity, risk factors, pathophysiology, clinical presentation, and diagnosis.. P&T.

[84] Surmeier D.J. (2018). Determinants of dopaminergic neuron loss in Parkinson’s disease.. FEBS J..

[85] Bose A., Beal M.F. (2016). Mitochondrial dysfunction in Parkinson’s disease.. J. Neurochem..

[86] Ammal Kaidery N., Thomas B. (2018). Current perspective of mitochondrial biology in Parkinson’s disease.. Neurochem. Int..

[87] Ryan B.J., Hoek S., Fon E.A., Wade-Martins R. (2015). Mitochondrial dysfunction and mitophagy in Parkinson’s: from familial to sporadic disease.. Trends Biochem. Sci..

[88] Exner N., Lutz A.K., Haass C., Winklhofer K.F. (2012). Mitochondrial dysfunction in Parkinson’s disease: molecular mechanisms and pathophysiological consequences.. EMBO J..

[89] Hattori N., Tanaka M., Ozawa T., Mizuno Y. (1991). Immunohistochemical studies on complexes I, II, III, and IV of mitochondria in parkinson’s disease.. Ann. Neurol..

[90] Subramaniam S.R., Chesselet M.F. (2013). Mitochondrial dysfunction and oxidative stress in Parkinson’s disease.. Prog. Neurobiol..

[91] Büeler H. (2009). Impaired mitochondrial dynamics and function in the pathogenesis of Parkinson’s disease.. Exp. Neurol..

[92] Santos D., Esteves A.R., Silva D.F., Januário C., Cardoso S.M. (2015). The impact of mitochondrial fusion and fission modulation in sporadic Parkinson’s disease.. Mol. Neurobiol..

[93] Van Laar V.S., Arnold B., Howlett E.H., Calderon M.J., St Croix C.M., Greenamyre J.T., Sanders L.H., Berman S.B. (2018). Evidence for compartmentalized axonal mitochondrial biogenesis: mitochondrial DNA replication increases in distal axons as an early response to Parkinson’s disease-relevant stress.. J. Neurosci..

[94] Zheng B., Liao Z., Locascio J.J., Lesniak K.A., Roderick S.S., Watt M.L., Eklund A.C., Zhang-James Y., Kim P.D., Hauser M.A., Grünblatt E., Moran L.B., Mandel S.A., Riederer P., Miller R.M., Federoff H.J., Wüllner U., Papapetropoulos S., Youdim M.B., Cantuti-Castelvetri I., Young A.B., Vance J.M., Davis R.L., Hedreen J.C., Adler C.H., Beach T.G., Graeber M.B., Middleton F.A., Rochet J.C., Scherzer C.R. (2010). PGC-1α, a potential therapeutic target for early intervention in Parkinson’s disease.. Sci. Transl. Med..

[95] Kraytsberg Y., Kudryavtseva E., McKee A.C., Geula C., Kowall N.W., Khrapko K. (2006). Mitochondrial DNA deletions are abundant and cause functional impairment in aged human substantia nigra neurons.. Nat. Genet..

[96] Parker W.D., Parks J.K., Swerdlow R.H. (2008). Complex I deficiency in Parkinson’s disease frontal cortex.. Brain Res..

[97] Pienaar I.S., Elson J.L., Racca C., Nelson G., Turnbull D.M., Morris C.M. (2013). Mitochondrial abnormality associates with type-specific neuronal loss and cell morphology changes in the pedunculopontine nucleus in Parkinson disease.. Am. J. Pathol..

[98] Schwarz T.L. (2013). Mitochondrial trafficking in neurons.. Cold Spring Harb. Perspect. Biol..

[99] Henchcliffe C., Beal M.F. (2008). Mitochondrial biology and oxidative stress in Parkinson disease pathogenesis.. Nat. Clin. Pract. Neurol..

[100] Pickrell A.M., Youle R.J. (2015). The roles of PINK1, parkin, and mitochondrial fidelity in Parkinson’s disease.. Neuron.

[101] Thomas B, Beal MF (2007). Parkinson's disease.. Hum Mol Genet..

[102] Trempe J.F., Fon E.A. (2013). Structure and Function of Parkin, PINK1, and DJ-1, the Three Musketeers of Neuroprotection.. Front. Neurol..

[103] Wang W., Wang X., Fujioka H., Hoppel C., Whone A.L., Caldwell M.A., Cullen P.J., Liu J., Zhu X. (2016). Parkinson’s disease–associated mutant VPS35 causes mitochondrial dysfunction by recycling DLP1 complexes.. Nat. Med..

[104] Rocha EM, De Miranda B, Sanders LH (2018). Alpha-synuclein: Pathology, mitochondrial dysfunction and neuroinflammation in Parkinson's disease.. Neurobiol Dis..

[105] Nakamura K., Nemani V.M., Azarbal F., Skibinski G., Levy J.M., Egami K., Munishkina L., Zhang J., Gardner B., Wakabayashi J., Sesaki H., Cheng Y., Finkbeiner S., Nussbaum R.L., Masliah E., Edwards R.H. (2011). Direct membrane association drives mitochondrial fission by the Parkinson disease-associated protein alpha-synuclein.. J. Biol. Chem..

[106] Mallach A., Weinert M., Arthur J., Gveric D., Tierney T.S., Alavian K.N. (2019). *Post mortem* examination of Parkinson’s disease brains suggests decline in mitochondrial biomass, reversed by deep brain stimulation of subthalamic nucleus.. FASEB J..

[107] Bekar L., Libionka W., Tian G.F., Xu Q., Torres A., Wang X., Lovatt D., Williams E., Takano T., Schnermann J., Bakos R., Nedergaard M. (2008). Adenosine is crucial for deep brain stimulation–mediated attenuation of tremor.. Nat. Med..

[108] Vosler P.S., Graham S.H., Wechsler L.R., Chen J. (2009). Mitochondrial targets for stroke: focusing basic science research toward development of clinically translatable therapeutics.. Stroke.

[109] Liu F., Lu J., Manaenko A., Tang J., Hu Q. (2018). Mitochondria in Ischemic Stroke: New Insight and Implications.. Aging Dis..

[110] Doyle K.P., Simon R.P., Stenzel-Poore M.P. (2008). Mechanisms of ischemic brain damage.. Neuropharmacology.

[111] Khoshnam S.E., Winlow W., Farzaneh M., Farbood Y., Moghaddam H.F. (2017). Pathogenic mechanisms following ischemic stroke.. Neurol. Sci..

[112] Jia J., Jin H., Nan D., Yu W., Huang Y. (2021). New insights into targeting mitochondria in ischemic injury.. Apoptosis.

[113] Ham P.B., Raju R. (2017). Mitochondrial function in hypoxic ischemic injury and influence of aging.. Prog. Neurobiol..

[114] Broughton B.R.S., Reutens D.C., Sobey C.G. (2009). Apoptotic mechanisms after cerebral ischemia.. Stroke.

[115] Zheng Z., Zhao H., Steinberg G.K., Yenari M.A. (2003). Cellular and molecular events underlying ischemia-induced neuronal apoptosis.. Drug News Perspect..

[116] Sims N.R., Muyderman H. (2010). Mitochondria, oxidative metabolism and cell death in stroke.. Biochim. Biophys. Acta Mol. Basis Dis..

[117] Galluzzi L., Kepp O., Kroemer G. (2012). Mitochondria: master regulators of danger signalling.. Nat. Rev. Mol. Cell Biol..

[118] Endo H., Kamada H., Nito C., Nishi T., Chan P.H. (2006). Mitochondrial translocation of p53 mediates release of cytochrome c and hippocampal CA1 neuronal death after transient global cerebral ischemia in rats.. J. Neurosci..

[119] Hares M.M., Downing R., Marsh J. (1980). Failure of metronidazole/penicillin oral prophylaxis to prevent amputation stump infection.. Lancet.

[120] Crack P.J., Taylor J.M. (2005). Reactive oxygen species and the modulation of stroke.. Free Radic. Biol. Med..

[121] Dharmasaroja P.A. (2016). Fluid Intake Related to Brain Edema in Acute Middle Cerebral Artery Infarction.. Transl. Stroke Res..

[122] Lee J.M., Grabb M.C., Zipfel G.J., Choi D.W. (2000). Brain tissue responses to ischemia.. J. Clin. Invest..

[123] Zhao H., Yenari M.A., Cheng D., Sapolsky R.M., Steinberg G.K. (2003). Bcl-2 overexpression protects against neuron loss within the ischemic margin following experimental stroke and inhibits cytochrome c translocation and caspase-3 activity.. J. Neurochem..

[124] Barsoum M.J., Yuan H., Gerencser A.A., Liot G., Kushnareva Y., Gräber S., Kovacs I., Lee W.D., Waggoner J., Cui J., White A.D., Bossy B., Martinou J.C., Youle R.J., Lipton S.A., Ellisman M.H., Perkins G.A., Bossy-Wetzel E. (2006). Nitric oxide-induced mitochondrial fission is regulated by dynamin-related GTPases in neurons.. EMBO J..

[125] Grohm J., Kim S-W., Mamrak U., Tobaben S., Cassidy-Stone A., Nunnari J., Plesnila N., Culmsee C. (2012). Inhibition of Drp1 provides neuroprotection *in vitro* and *in vivo.*. Cell Death Differ..

[126] Zhang L., He Z., Zhang Q., Wu Y., Yang X., Niu W., Hu Y., Jia J. (2014). Exercise pretreatment promotes mitochondrial dynamic protein OPA1 expression after cerebral ischemia in rats.. Int. J. Mol. Sci..

[127] Song M., Zhou Y., Fan X. (2022). Mitochondrial quality and quantity control: mitophagy is a potential therapeutic target for ischemic stroke.. Mol. Neurobiol..

[128] Di Y., He Y.L., Zhao T., Huang X., Wu K.W., Liu S.H., Zhao Y.Q., Fan M., Wu L.Y., Zhu L.L. (2015). Methylene blue reduces acute cerebral ischemic injury *via* the induction of mitophagy.. Mol. Med..

[129] Liang J., Wang C., Zhang H., Huang J., Xie J., Chen N. (2021). Exercise-induced benefits for Alzheimer’s disease by stimulating mitophagy and improving mitochondrial function.. Front. Aging Neurosci..

[130] Raefsky S.M., Mattson M.P. (2017). Adaptive responses of neuronal mitochondria to bioenergetic challenges: Roles in neuroplasticity and disease resistance.. Free Radic. Biol. Med..

[131] Escribano L., Simón A.M., Gimeno E., Cuadrado-Tejedor M., López de Maturana R., García-Osta A., Ricobaraza A., Pérez-Mediavilla A., Del Río J., Frechilla D. (2010). Rosiglitazone rescues memory impairment in Alzheimer’s transgenic mice: mechanisms involving a reduced amyloid and tau pathology.. Neuropsychopharmacology.

[132] Searcy J.L., Phelps J.T., Pancani T., Kadish I., Popovic J., Anderson K.L., Beckett T.L., Murphy M.P., Chen K.C., Blalock E.M., Landfield P.W., Porter N.M., Thibault O. (2012). Long-term pioglitazone treatment improves learning and attenuates pathological markers in a mouse model of Alzheimer’s disease.. J. Alzheimers Dis..

[133] Pinto M., Nissanka N., Peralta S., Brambilla R., Diaz F., Moraes C.T. (2016). Pioglitazone ameliorates the phenotype of a novel Parkinson’s disease mouse model by reducing neuroinflammation.. Mol. Neurodegener..

[134] Zhao Y., Lützen U., Gohlke P., Jiang P., Herdegen T., Culman J. (2021). Neuroprotective and antioxidative effects of pioglitazone in brain tissue adjacent to the ischemic core are mediated by PI3K/Akt and Nrf2/ARE pathways.. J. Mol. Med. (Berl.).

[135] Luo Y., Yin W., Signore A.P., Zhang F., Hong Z., Wang S., Graham S.H., Chen J. (2006). Neuroprotection against focal ischemic brain injury by the peroxisome proliferator-activated receptor-γ agonist rosiglitazone.. J. Neurochem..

[136] (2015). Pioglitazone in early Parkinson’s disease: a phase 2, multicentre, double-blind, randomised trial.. Lancet Neurol..

[137] Risner M.E., Saunders A.M., Altman J F B., Ormandy G.C., Craft S., Foley I.M., Zvartau-Hind M.E., Hosford D.A., Roses A.D. (2006). Efficacy of rosiglitazone in a genetically defined population with mild-to-moderate Alzheimer’s disease.. Pharmacogenomics J..

[138] Beal M.F., Oakes D., Shoulson I., Henchcliffe C., Galpern W.R., Haas R., Juncos J.L., Nutt J.G., Voss T.S., Ravina B., Shults C.M., Helles K., Snively V., Lew M.F., Griebner B., Watts A., Gao S., Pourcher E., Bond L., Kompoliti K., Agarwal P., Sia C., Jog M., Cole L., Sultana M., Kurlan R., Richard I., Deeley C., Waters C.H., Figueroa A., Arkun A., Brodsky M., Ondo W.G., Hunter C.B., Jimenez-Shahed J., Palao A., Miyasaki J.M., So J., Tetrud J., Reys L., Smith K., Singer C., Blenke A., Russell D.S., Cotto C., Friedman J.H., Lannon M., Zhang L., Drasby E., Kumar R., Subramanian T., Ford D.S., Grimes D.A., Cote D., Conway J., Siderowf A.D., Evatt M.L., Sommerfeld B., Lieberman A.N., Okun M.S., Rodriguez R.L., Merritt S., Swartz C.L., Martin W.R.W., King P., Stover N., Guthrie S., Watts R.L., Ahmed A., Fernandez H.H., Winters A., Mari Z., Dawson T.M., Dunlop B., Feigin A.S., Shannon B., Nirenberg M.J., Ogg M., Ellias S.A., Thomas C.A., Frei K., Bodis-Wollner I., Glazman S., Mayer T., Hauser R.A., Pahwa R., Langhammer A., Ranawaya R., Derwent L., Sethi K.D., Farrow B., Prakash R., Litvan I., Robinson A., Sahay A., Gartner M., Hinson V.K., Markind S., Pelikan M., Perlmutter J.S., Hartlein J., Molho E., Evans S., Adler C.H., Duffy A., Lind M., Elmer L., Davis K., Spears J., Wilson S., Leehey M.A., Hermanowicz N., Niswonger S., Shill H.A., Obradov S., Rajput A., Cowper M., Lessig S., Song D., Fontaine D., Zadikoff C., Williams K., Blindauer K.A., Bergholte J., Propsom C.S., Stacy M.A., Field J., Mihaila D., Chilton M., Uc E.Y., Sieren J., Simon D.K., Kraics L., Silver A., Boyd J.T., Hamill R.W., Ingvoldstad C., Young J., Thomas K., Kostyk S.K., Wojcieszek J., Pfeiffer R.F., Panisset M., Beland M., Reich S.G., Cines M., Zappala N., Rivest J., Zweig R., Lumina L.P., Hilliard C.L., Grill S., Kellermann M., Tuite P., Rolandelli S., Kang U.J., Young J., Rao J., Cook M.M., Severt L., Boyar K. (2014). A randomized clinical trial of high-dosage coenzyme Q10 in early Parkinson disease: no evidence of benefit.. JAMA Neurol..

[139] Xi Y., Feng D., Tao K., Wang R., Shi Y., Qin H., Murphy M.P., Yang Q., Zhao G. (2018). MitoQ protects dopaminergic neurons in a 6-OHDA induced PD model by enhancing Mfn2-dependent mitochondrial fusion *via* activation of PGC-1α.. Biochim. Biophys. Acta Mol. Basis Dis..

[140] Ghosh A., Langley M.R., Harischandra D.S., Neal M.L., Jin H., Anantharam V., Joseph J., Brenza T., Narasimhan B., Kanthasamy A., Kalyanaraman B., Kanthasamy A.G. (2016). Mitoapocynin treatment protects against neuroinflammation and dopaminergic neurodegeneration in a preclinical animal model of Parkinson’s Disease.. J. Neuroimmune Pharmacol..

[141] Manczak M., Mao P., Calkins M.J., Cornea A., Reddy A.P., Murphy M.P., Szeto H.H., Park B., Reddy P.H. (2010). Mitochondria-targeted antioxidants protect against amyloid-beta toxicity in Alzheimer’s disease neurons.. J. Alzheimers Dis..

[142] Silachev D., Plotnikov E., Pevzner I., Zorova L., Balakireva A., Gulyaev M., Pirogov Y., Skulachev V., Zorov D. (2018). Neuroprotective effects of mitochondria-targeted plastoquinone in a rat model of neonatal hypoxic–ischemic brain injury.. Molecules.

[143] Snow B.J., Rolfe F.L., Lockhart M.M., Frampton C.M., O’Sullivan J.D., Fung V., Smith R.A.J., Murphy M.P., Taylor K.M. (2010). A double-blind, placebo-controlled study to assess the mitochondria-targeted antioxidant MitoQ as a disease-modifying therapy in Parkinson’s disease.. Mov. Disord..

[144] Shuaib A., Lees K.R., Lyden P., Grotta J., Davalos A., Davis S.M., Diener H.C., Ashwood T., Wasiewski W.W., Emeribe U. (2007). NXY-059 for the treatment of acute ischemic stroke.. N. Engl. J. Med..

[145] Wang Q.M., Xu Y.Y., Liu S., Ma Z.G. (2017). Isradipine attenuates MPTP-induced dopamine neuron degeneration by inhibiting up-regulation of L-type calcium channels and iron accumulation in the substantia nigra of mice.. Oncotarget.

[146] Gong L., Zhang Q.L., Zhang N., Hua W.Y., Huang Y.X., Di P.W., Huang T., Xu X.S., Liu C.F., Hu L.F., Luo W.F. (2012). Neuroprotection by urate on 6-OHDA-lesioned rat model of Parkinson’s disease: Linking to Akt/GSK3β signaling pathway.. J. Neurochem..

[147] Baek S.H., Park S.J., Jeong J.I., Kim S.H., Han J., Kyung J.W., Baik S.H., Choi Y., Choi B.Y., Park J.S., Bahn G., Shin J.H., Jo D.S., Lee J.Y., Jang C.G., Arumugam T.V., Kim J., Han J.W., Koh J.Y., Cho D.H., Jo D.G. (2017). Inhibition of Drp1 ameliorates synaptic depression, Aβ deposition, and cognitive impairment in an Alzheimer’s Disease model.. J. Neurosci..

[148] Filichia E., Hoffer B., Qi X., Luo Y. (2016). Inhibition of Drp1 mitochondrial translocation provides neural protection in dopaminergic system in a Parkinson’s disease model induced by MPTP.. Sci. Rep..

[149] Flippo K.H., Lin Z., Dickey A.S., Zhou X., Dhanesha N.A., Walters G.C., Liu Y., Merrill R.A., Meller R., Simon R.P., Chauhan A.K., Usachev Y.M., Strack S. (2020). Deletion of a neuronal Drp1 activator protects against cerebral ischemia.. J. Neurosci..

[150] Santos R.X., Correia S.C., Carvalho C., Cardoso S., Santos M.S., Moreira P.I. (2011). Mitophagy in neurodegeneration: An opportunity for therapy?. Curr. Drug Targets.

[151] Zhang L., Dai L., Li D. (2021). Mitophagy in neurological disorders.. J. Neuroinflammation.

[152] Nascimento-dos-Santos G., de-Souza-Ferreira E., Linden R., Galina A., Petrs-Silva H. (2021). Mitotherapy: Unraveling a promising treatment for disorders of the central nervous system and other systemic conditions.. Cells.

[153] Qin Y., Jiang X., Yang Q., Zhao J., Zhou Q., Zhou Y. (2021). The functions, methods, and mobility of mitochondrial transfer between cells.. Front. Oncol..

[154] Jackson M.V., Morrison T.J., Doherty D.F., McAuley D.F., Matthay M.A., Kissenpfennig A., O’Kane C.M., Krasnodembskaya A.D. (2016). Mitochondrial transfer *via* tunneling nanotubes is an important mechanism by which mesenchymal stem cells enhance macrophage phagocytosis in the *in vitro* and *in vivo* models of ARDS.. Stem Cells.

[155] Jiang D., Gao F., Zhang Y., Wong D.S.H., Li Q., Tse H., Xu G., Yu Z., Lian Q. (2016). Mitochondrial transfer of mesenchymal stem cells effectively protects corneal epithelial cells from mitochondrial damage.. Cell Death Dis..

[156] Islam M.N., Das S.R., Emin M.T., Wei M., Sun L., Westphalen K., Rowlands D.J., Quadri S.K., Bhattacharya S., Bhattacharya J. (2012). Mitochondrial transfer from bone-marrow–derived stromal cells to pulmonary alveoli protects against acute lung injury.. Nat. Med..

[157] Hayakawa K., Esposito E., Wang X., Terasaki Y., Liu Y., Xing C., Ji X., Lo E.H. (2016). Transfer of mitochondria from astrocytes to neurons after stroke.. Nature.

[158] Elliott R.L., Jiang X.P., Head J.F. (2012). Mitochondria organelle transplantation: introduction of normal epithelial mitochondria into human cancer cells inhibits proliferation and increases drug sensitivity.. Breast Cancer Res. Treat..

[159] Moskowitzova K., Shin B., Liu K., Ramirez-Barbieri G., Guariento A., Blitzer D., Thedsanamoorthy J.K., Yao R., Snay E.R., Inkster J.A.H., Orfany A., Zurakowski D., Cowan D.B., Packard A.B., Visner G.A., del Nido P.J., McCully J.D. (2019). Mitochondrial transplantation prolongs cold ischemia time in murine heart transplantation.. J. Heart Lung Transplant..

[160] Hayashida K., Takegawa R., Shoaib M., Aoki T., Choudhary R.C., Kuschner C.E., Nishikimi M., Miyara S.J., Rolston D.M., Guevara S., Kim J., Shinozaki K., Molmenti E.P., Becker L.B. (2021). Mitochondrial transplantation therapy for ischemia reperfusion injury: A systematic review of animal and human studies.. J. Transl. Med..

[161] Guariento A., Piekarski B.L., Doulamis I.P., Blitzer D., Ferraro A.M., Harrild D.M., Zurakowski D., del Nido P.J., McCully J.D., Emani S.M. (2021). Autologous mitochondrial transplantation for cardiogenic shock in pediatric patients following ischemia-reperfusion injury.. J. Thorac. Cardiovasc. Surg..

[162] Doulamis I.P., Guariento A., Duignan T., Orfany A., Kido T., Zurakowski D., del Nido P.J., McCully J.D. (2020). Mitochondrial transplantation for myocardial protection in diabetic hearts.. Eur. J. Cardiothorac. Surg..

[163] Robicsek O., Ene H.M., Karry R., Ytzhaki O., Asor E., McPhie D., Cohen B.M., Ben-Yehuda R., Weiner I., Ben-Shachar D. (2018). Isolated mitochondria transfer improves neuronal differentiation of schizophrenia-derived induced pluripotent stem cells and rescues deficits in a rat model of the disorder.. Schizophr. Bull..

[164] Clark M.A., Shay J.W. (1982). Mitochondrial transformation of mammalian cells.. Nature.

[165] Davis C.O., Kim K.Y., Bushong E.A., Mills E.A., Boassa D., Shih T., Kinebuchi M., Phan S., Zhou Y., Bihlmeyer N.A., Nguyen J.V., Jin Y., Ellisman M.H., Marsh-Armstrong N. (2014). Transcellular degradation of axonal mitochondria.. Proc. Natl. Acad. Sci. USA.

[166] Forner F., Foster L.J., Campanaro S., Valle G., Mann M. (2006). Quantitative proteomic comparison of rat mitochondria from muscle, heart, and liver.. Mol. Cell. Proteomics.

[167] Cogswell A.M., Stevens R.J., Hood D.A. (1993). Properties of skeletal muscle mitochondria isolated from subsarcolemmal and intermyofibrillar regions.. Am. J. Physiol. Cell Physiol..

[168] Preble J.M., Pacak C.A., Kondo H., MacKay A.A., Cowan D.B., McCully J.D. (2014). Rapid isolation and purification of mitochondria for transplantation by tissue dissociation and differential filtration.. J. Vis. Exp..

[169] Chang J.C., Wu S.L., Liu K.H., Chen Y.H., Chuang C.S., Cheng F.C., Su H.L., Wei Y.H., Kuo S.J., Liu C.S. (2016). Allogeneic/xenogeneic transplantation of peptide-labeled mitochondria in Parkinson’s disease: Restoration of mitochondria functions and attenuation of 6-hydroxydopamine–induced neurotoxicity.. Transl. Res..

[170] Gollihue J.L., Patel S.P., Mashburn C., Eldahan K.C., Sullivan P.G., Rabchevsky A.G. (2017). Optimization of mitochondrial isolation techniques for intraspinal transplantation procedures.. J. Neurosci. Methods.

[171] Masuzawa A., Black K.M., Pacak C.A., Ericsson M., Barnett R.J., Drumm C., Seth P., Bloch D.B., Levitsky S., Cowan D.B., McCully J.D. (2013). Transplantation of autologously derived mitochondria protects the heart from ischemia-reperfusion injury.. Am. J. Physiol. Heart Circ. Physiol..

[172] Ramirez-Barbieri G., Moskowitzova K., Shin B., Blitzer D., Orfany A., Guariento A., Iken K., Friehs I., Zurakowski D., del Nido P.J., McCully J.D. (2019). Alloreactivity and allorecognition of syngeneic and allogeneic mitochondria.. Mitochondrion.

[173] Pollara J., Edwards R.W., Lin L., Bendersky V.A., Brennan T.V. (2018). Circulating mitochondria in deceased organ donors are associated with immune activation and early allograft dysfunction.. JCI Insight.

[174] Krysko D.V., Agostinis P., Krysko O., Garg A.D., Bachert C., Lambrecht B.N., Vandenabeele P. (2011). Emerging role of damage-associated molecular patterns derived from mitochondria in inflammation.. Trends Immunol..

[175] Bertero E., O’Rourke B., Maack C. (2020). Mitochondria do not survive calcium overload during transplantation.. Circ. Res..

[176] Al Amir Dache Z., Otandault A., Tanos R., Pastor B., Meddeb R., Sanchez C., Arena G., Lasorsa L., Bennett A., Grange T., El Messaoudi S., Mazard T., Prevostel C., Thierry A.R. (2020). Blood contains circulating cell‐free respiratory competent mitochondria.. FASEB J..

[177] Chang J.C., Hoel F., Liu K.H., Wei Y.H., Cheng F.C., Kuo S.J., Tronstad K.J., Liu C.S. (2017). Peptide-mediated delivery of donor mitochondria improves mitochondrial function and cell viability in human cybrid cells with the MELAS A3243G mutation.. Sci. Rep..

[178] Wu S., Zhang A., Li S., Chatterjee S., Qi R., Segura-Ibarra V., Ferrari M., Gupte A., Blanco E., Hamilton D.J. (2018). Polymer functionalization of isolated mitochondria for cellular transplantation and metabolic phenotype alteration.. Adv. Sci. (Weinh.).

[179] Picone P., Porcelli G., Bavisotto C.C., Nuzzo D., Galizzi G., Biagio P.L.S., Bulone D., Di Carlo M. (2021). Synaptosomes: New vesicles for neuronal mitochondrial transplantation.. J. Nanobiotechnology.

[180] Guariento A., Doulamis I.P., Duignan T., Kido T., Regan W.L., Saeed M.Y., Hoganson D.M., Emani S.M., Fynn-Thompson F., Matte G.S., del Nido P.J., McCully J.D. (2020). Mitochondrial transplantation for myocardial protection in ex-situ‒perfused hearts donated after circulatory death.. J. Heart Lung Transplant..

[181] Adlimoghaddam A., Benson T., Albensi B.C. (2022). Mitochondrial transfusion improves mitochondrial function through up-regulation of mitochondrial complex II protein subunit SDHB in the hippocampus of aged mice.. Mol. Neurobiol..

[182] Bobkova N.V., Zhdanova D.Y., Belosludtseva N.V., Penkov N.V., Mironova G.D. (2022). Intranasal administration of mitochondria improves spatial memory in olfactory bulbectomized mice.. Exp. Biol. Med. (Maywood).

[183] Nakamura Y., Lo E.H., Hayakawa K. (2020). Placental mitochondria therapy for cerebral ischemia-reperfusion injury in mice.. Stroke.

[184] Pourmohammadi-Bejarpasi Z., Roushandeh A.M., Saberi A., Rostami M.K., Toosi S.M.R., Jahanian-Najafabadi A., Tomita K., Kuwahara Y., Sato T., Roudkenar M.H. (2020). Mesenchymal stem cells-derived mitochondria transplantation mitigates I/R-induced injury, abolishes I/R-induced apoptosis, and restores motor function in acute ischemia stroke rat model.. Brain Res. Bull..

[185] Huang P.J., Kuo C.C., Lee H.C., Shen C.I., Cheng F.C., Wu S.F., Chang J.C., Pan H.C., Lin S.Z., Liu C.S., Su H.L. (2016). Transferring Xenogenic mitochondria provides neural protection against ischemic stress in ischemic rat brains.. Cell Transplant..

[186] Xie Q., Zeng J., Zheng Y., Li T., Ren J., Chen K., Zhang Q., Xie R., Xu F., Zhu J. (2021). Mitochondrial transplantation attenuates cerebral ischemia-reperfusion injury: Possible involvement of mitochondrial component separation.. Oxid. Med. Cell. Longev..

